# Enhanced Magnetic Resonance Imaging–Based Knee Cartilage Segmentation Using a Swin-UNet Conditional Generative Adversarial Network: Development and Validation Study

**DOI:** 10.2196/86155

**Published:** 2026-03-02

**Authors:** Jun Young Park, Ji-Hoon Nam, Shakhboz Abdigapporov, Jong-Keun Kim, Yong-Gon Koh, Byung Woo Cho, Hyuck Min Kwon, Kwan Kyu Park, Kyoung-Tak Kang

**Affiliations:** 1 Department of Orthopaedic Surgery Yonsei University College of Medicine Yongin Severance Hospital Yongin-si, Gyeonggi-do Republic of Korea; 2 Department of Mechanical Engineering Yonsei University Seoul, Seoul Republic of Korea; 3 Clevion Co., Ltd. Seoul Republic of Korea; 4 Skyve R&D LAB Skyve Co., Ltd. Seoul Republic of Korea; 5 Department of Orthopaedic Surgery HeungK Hospital Seoul Republic of Korea; 6 Department of Orthopaedic Surgery Joint Reconstruction Center Yonsei Sarang Hospital Seoul Republic of Korea; 7 Department of Orthopaedic Surgery Yonsei University College of Medicine Gangnam Severance Hospital Seoul Republic of Korea; 8 Department of Orthopaedic Surgery Yonsei University College of Medicine Severance Hospital Seoul Republic of Korea

**Keywords:** cartilage, deep learning, generative adversarial network, knee cartilage segmentation, magnetic resonance imaging, MRI, segmentation, swim-UNet

## Abstract

**Background:**

Accurate segmentation of cartilage from magnetic resonance imaging (MRI) is crucial for the diagnosis and surgical planning of knee osteoarthritis. However, manual segmentation is time-consuming, and conventional computed tomography–based surgical systems are limited by their inability to visualize cartilage.

**Objective:**

This study aimed to develop a clinically targeted deep learning framework, the Swin-UNet conditional generative adversarial network (cGAN), for the automatic segmentation of femoral and tibial cartilage in MRI. We then evaluated its performance against conventional UNet, UNet cGAN, and Swin-UNet baseline models.

**Methods:**

Our dataset comprised 232 knee MRI scans. We conducted quantitative experiments on the proposed Swin-UNet cGAN model and compared the results with those of widely used UNet, UNet cGAN, and Swin-UNet models for femoral and tibial cartilage segmentation, using the Dice similarity coefficient, mean intersection over union, 95th percentile Hausdorff distance, and average symmetric surface distance. All performance metrics were statistically analyzed. In addition, the performance of the Swin-UNet cGAN model was evaluated on an external validation dataset.

**Results:**

The proposed Swin-UNet cGAN achieved the highest mean Dice similarity coefficient and intersection over union scores for both femoral and tibial cartilage segmentation, demonstrating performance statistically comparable to the best-performing baseline (UNet) in the tibia. Regarding distance metrics (average symmetric surface distance and 95th percentile Hausdorff distance), the proposed model significantly outperformed all baselines in the tibia while achieving results comparable to the UNet cGAN in the femur. It also maintained consistently high segmentation performance on both the internal test set and an external validation dataset.

**Conclusions:**

These findings indicate that the proposed Swin-UNet cGAN achieves more accurate knee cartilage segmentation than UNet, UNet cGAN, and Swin-UNet, particularly in terms of boundary accuracy, while maintaining promising generalizability performance across both internal testing and external validation cohorts. This MRI-based deep learning approach addresses critical limitations of computed tomography–based patient-specific instrumentation systems by providing cartilage visualization, potentially improving surgical precision and outcomes in total knee arthroplasty.

## Introduction

Knee joint osteoarthritis is a progressive degenerative disorder affecting the articular cartilage, underlying subchondral bone, and surrounding periarticular tissues. It presents with pain, stiffness, and reduced mobility, ranking among the leading causes of disability in adults [1,2]. Timely identification and precise quantification of alterations in cartilage structure and composition are essential for optimal management of knee osteoarthritis [3].

An extensive array of imaging modalities is used in clinical and research settings to deepen understanding of osteoarthritis pathogenesis [4]. Magnetic resonance imaging (MRI) offers superior visualization of cartilage structure and is the modality of choice for assessing cartilage damage and morphological alterations. In addition, MRI provides a noninvasive, radiation-free examination and excels at evaluating overall knee pathology, owing to its superior soft-tissue contrast relative to X-ray and computed tomography (CT) [5].

Quantitative analysis of cartilage morphology depends on accurate medical-image segmentation and visualization of the articular cartilage, with subsequent 3D reconstruction enabling measurement of thickness, volume, and surface area [6]. However, this process is complex and time-consuming, as each image slice must be segmented individually using manual segmentation software [7]. In recent years, deep learning (DL)–based artificial intelligence models, particularly convolutional neural network (CNN) architectures, have been adopted as a cutting-edge solution for knee cartilage segmentation and osteoarthritis diagnosis [8]. CNN-based models have shown strong potential for accurately segmenting cartilage and bone across imaging modalities [9].

Toit et al [9] studied a modified UNet algorithm to automatically segment the femoral articular cartilage in 3D ultrasound images of healthy volunteer knees. Wang et al [10] proposed a novel network, Patch Attention UNet, to enhance knee cartilage segmentation by applying an attention mechanism; it was evaluated on multiple datasets. Recently, generative adversarial networks (GANs) have captivated the research community, with several studies demonstrating that GAN-generated delineations can match manual tracings and materially enhance segmentation performance [11]. Gaj et al [11] developed a conditional generative adversarial network (cGAN) model built on the UNet architecture that delivers fully automated, highly accurate segmentation of knee cartilage and meniscus.

Therefore, our objective was to develop a Swin-UNet cGAN, a tailored adversarial framework merging a hierarchical Swin-UNet generator with a pixel-wise CNN discriminator for 2D segmentation of knee joint cartilage, and to evaluate its performance on both an internal test set and an external validation dataset. We compared the segmentation performance of our proposed model with traditional UNet, UNet cGAN, and Swin-UNet models using the Dice similarity coefficient (DSC), mean Intersection over Union (IoU), average symmetric surface distance (ASSD), and 95th percentile Hausdorff distance (HD95).

We hypothesized that the Swin-UNet cGAN model would achieve the highest performance in segmenting femoral and tibial cartilage.

## Methods

### Ethical Considerations

This study was approved by the Institutional Review Board (IRB) of Yonsei Sarang Hospital (IRB number 2025-11-002) and Yongin Severance Hospital (IRB number 9-2025-0189). The requirement for informed consent was waived by the IRB owing to the retrospective nature of this study and the use of deidentified imaging data. All procedures involving human participants were conducted in accordance with institutional ethical standards and approved by the relevant IRBs. All patient data were fully anonymized prior to analysis, with no possibility of individual patient identification. Patients received no compensation for the use of their deidentified MRI data in this research. No identifiable patient information or images are included in this manuscript or its supplementary materials.

### Dataset

The internal dataset was collected from Yonsei Sarang Hospital and consisted of 232 MRI scans from patients with knee osteoarthritis scheduled for patient-specific instrument (PSI)–guided total knee arthroplasty (TKA) from October 2023 through December 2024. All internal examinations were performed on a 3.0 T system (uMR 770; United Imaging Healthcare) using a 3D high-resolution T2*-weighted gradient-echo sequence (repetition time [TR]/echo time [TE]=17/9.3 ms; flip angle=25°), with a 20-cm field of view and 1-mm thick sagittal slices oriented relative to the posterior femoral condyles. This imaging protocol is commonly used when fabricating PSI [12]. Each scan comprised 90-110 slices. A medical imaging specialist manually segmented cartilage on every slice. The 232 scans were randomly split at the case level: a total of 162 (70%) scans were assigned to the training set, 35 (15%) scans to the validation set, and 35 (15%) scans to the testing set. For model development, the original 512 × 512 pixel images and their corresponding masks were down-sampled to 224 × 224 pixels.

For external validation, an independent dataset was obtained from Yongin Severance Hospital, consisting of 25 consecutive PSI-guided TKA cases. All external validation knee MRI examinations were performed on a 3.0 T system (SIGNA Premier; GE Healthcare) with a 20-cm field of view and 2-mm thick sagittal slices oriented relative to the posterior femoral condyles. TR and TE were automatically adjusted by the scanner to optimize image quality, with nominal target values of TR/TE=30/11 ms.

### Swin-UNet–Based cGAN Model

GAN-based networks usually consist of 2 parts: a generator model that produces synthetic samples from noise with a prior distribution and a discriminator model that acts as a binary classifier to differentiate real data from generated synthetic samples. In this study, the widely used cGAN [13] was adopted for MRI knee bone and cartilage segmentation. In this work, the generator is a mapping function from a source domain image into a segmentation mask, rather than mapping noise to real data as in the original GAN model, and predicts a manual segmentation mask. The cGAN-based model uses the discriminator to judge the quality of segmentation given the source image from the dataset. The binary classifier distinguishes the differences between the manual segmentation mask and the synthetic segmentation mask generated by the generator model, designating them as real and fake samples, respectively. This study introduces a Swin-UNet–based cGAN for knee MRI segmentation [14] to create synthetic masks and a PatchGAN discriminator [15] that learns discriminative features to distinguish generated masks from manual annotations.

### Generator of the Swin-UNet–Based cGAN Model

**Figure 1:**
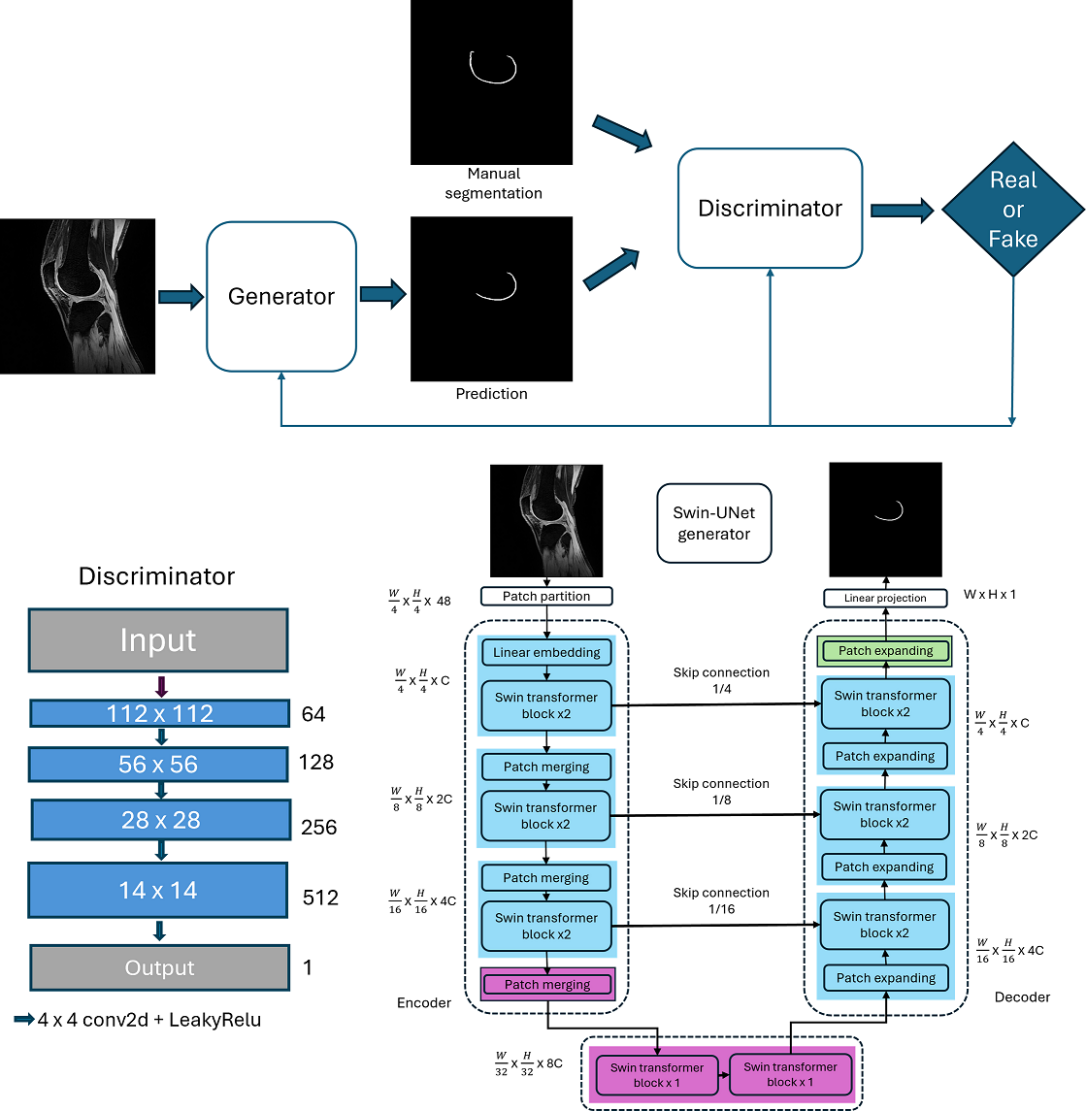
Overall architecture of the proposed network.

The generator of the knee cartilage segmentation model is a hierarchical Swin Transformer architecture integrated into a UNet-like encoder-decoder structure, as shown in Figure 1. The input image size and patch size are set as 224 × 224 and 4, respectively. The C-dimensional (C=32) tokenized inputs with a resolution of H/4 × W/4 are fed into 2 consecutive Swin Transformer blocks (using a 7 × 7 attention window) to perform representation learning, and the feature dimension and resolution remain unchanged. The patch merging layer reduces the number of tokens (down-samples by 2×) and increases the feature dimension to 2× the original dimension. The encoder repeats this procedure 3 times. The patch merging layer in the encoder divides the input patches into 4 parts and concatenates them together. The feature resolution is down-sampled by 2×, and because the concatenate operation increases the feature dimension by 4×, a linear layer is applied on the concatenated features to unify the feature dimension to 2× the original dimension. The bottleneck consists of 2 Swin Transformer blocks and is used to learn deep feature representation. In the bottleneck, the feature resolution and dimension remain unchanged.

The decoder has a symmetrical architecture to the encoder, based on the Swin Transformer blocks. The patch expanding layer in the decoder is used to up-sample the extracted deep features. The patch expanding layer reshapes the feature maps of adjacent dimensions into higher-resolution feature maps, up-sampling by 2×, and reduces the feature dimension to half of the original dimension.

Skip connections are used to fuse the multiscale features from the encoder with the up-sampled features. Both shallow and deep features are concatenated together to reduce the loss of spatial information caused by down-sampling.

### Discriminator of the Swin-UNet–Based cGAN Model

The discriminator of the proposed model is a PixelGAN-style convolutional binary classifier of “fake” predicted segmented masks and “real” manually segmented masks. The network input has 2 channels (image + mask) and spatial size H × W. The discriminator network architecture consists of four 4 × 4 convolutional layers with LeakyRelu activation function. Normalization is applied to stabilize the training samples, and Dropout2d prevents the discriminator from overpowering the generator in the early stages and encourages robust feature learning. The final layer is a 4 × 4 stride convolution, which produces a grid of real or fake logits. With 224 × 224 input, the discriminator produces a 13 × 13 authenticity map.

In the proposed model, a linearly increasing adversarial weight schedule was used, with λ starting at 0 after pretraining and gradually increasing to 1 × 10^–4^ over the GAN training epochs. This prevented gradient shocks and was selected as the most stable λ based on validation stability. Prior to selecting λ, larger values of 5 × 10^–4^ and 1 × 10^–3^ were empirically explored; although these values increased the adversarial influence, they led to unstable training.

### Loss Function of the Swin-UNet–Based cGAN Model

The proposed knee cartilage segmentation model uses a combination of supervised segmentation loss and adversarial losses. If segmentation performance mainly relies on adversarial loss, the output segmentation mask might not always share the same global structure as the manually segmented mask [16-19]. For traditional segmentation losses, the DSC and cross-entropy can be applied as loss functions for the generator network to improve performance by decreasing the distance between generated “fake” and manually segmented “real” masks. The proposed model uses multiple loss functions during training because the training experiments were conducted in multiple stages: generator model pretraining for segmentation mask predictions and overall training of the developed Swin-UNet cGAN–based knee segmentation model. During both the pretraining and overall model training phases, binary cross-entropy (BCE) with logits loss 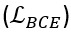 is adopted as the primary segmentation loss.


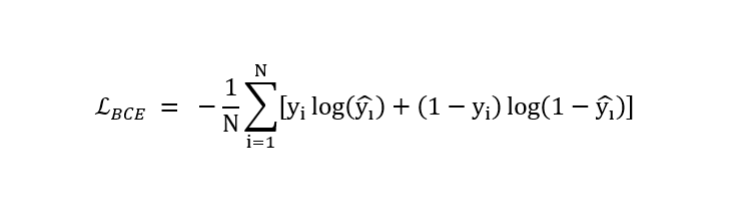


where yi is the ground truth mask of the real samples and 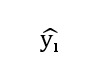 is the mask predicted by the generator network. For adversarial loss, a hinge loss formulation is applied to improve training stability.


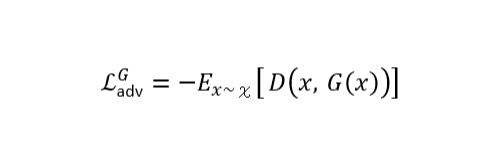


The discriminator network is trained using hinge loss to improve classification of real and fake sample data. For real mask samples, the discriminator is set to output values greater than 1; for fake samples, output are less than –1. The overall loss is computed as the mean of the 2 hinge components. The overall discriminator loss can be written as follows.


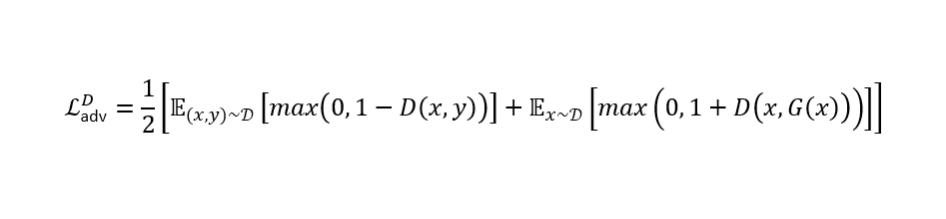


In the proposed model training, a gradient penalty was implemented to stabilize training by penalizing deviations in gradient norm from 1. Finally, the generator’s overall loss is composed of the total segmentation loss and a weighted adversarial loss.


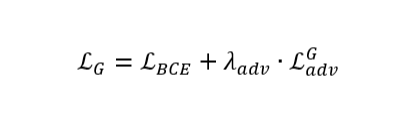


For the proposed model training, the Adam optimizer was used with a learning rate of 1 × 10^–4^ for the transformer-based generator and 5 × 10^–5^ for the patch-based discriminator networks, with a weight decay of 1 × 10^–5^. To maximize the prediction results of the segmentation model, the generator was first pretrained for 30 epochs using only the segmentation loss (BCE with logits loss). After pretraining, adversarial training was performed for 40 epochs using a combination of segmentation and adversarial losses. During overall model training, adversarial weight λ_adv_ increased linearly until it reached the final value of 1 × 10^–4^. Training of all experimented models was performed using an NVIDIA GeForce RTX 4090 24 GB graphics processing unit.

### Evaluation Metrics

The performance of the knee cartilage segmentation models was compared with that of the Swin-UNet segmentation model, the UNet-based GAN segmentation model [11], and the traditional CNN-based UNet model using the DSC, IoU, HD95, and ASSD. The DSC represent the overlap between the predicted and ground truth masks. A DSC of 1 (or 100%) represents perfect overlap, whereas a value of 0 indicates no overlap between the ground truth and predicted masks.


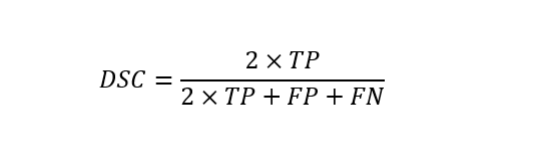


The mean IoU metric is calculated by dividing the area of intersection of the predicted and ground truth masks by the area of the union of 2 masks.


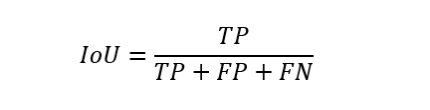


The Hausdorff distance (HD) measures the extent to which 2 subsets of a metric space differ. Because HD is sensitive to outliers, the 95th percentile (HD95) is frequently used instead. By considering the 95th percentile of distances, HD95 provides a more robust measure of boundary conformity, effectively ignoring the most extreme 5% of outliers.


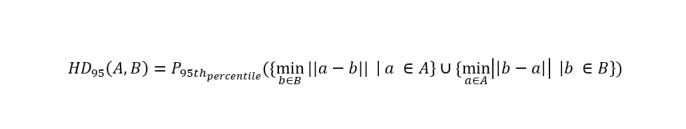


where A and B denote the sets of boundary points for the predicted and ground truth segmentations, respectively; ||a – b|| represents the Euclidean distance between points a and b; and P_95_ denotes the 95th percentile of the accumulated distances.

The ASSD measures the average distance between boundary points, making it highly sensitive to the precise alignment of contours.


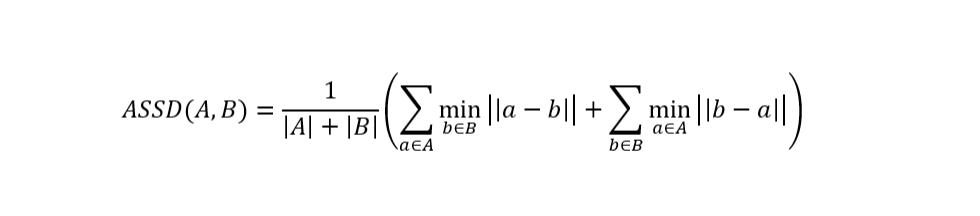


where |A| and |B| denote the cardinality of the boundary sets A and B, respectively. All surface metrics (ASSD and HD95) were computed on 224 × 224 masks, the same resolution used for training and inference. Distances were converted to millimeters using the effective in-plane spacing of 0.89 mm/pixel.

### Reliability of Manual Segmentation

For consistent and reliable manual labeling of our dataset, DSC and IoU metrics were calculated for intra- and interrater comparisons across femur and tibia segmentations. Two experienced manual data labeling experts, rater A and rater B, segmented the entire dataset. For reliability assessment, MRI data from 30 randomly selected patients were labeled a second time by both raters at a different time of day than the first labeling session. For the final training and test datasets, segmentation masks created by a single expert rater were used for each slice.

### Ablation Study

To empirically validate the architectural decisions and hyperparameter configurations of the proposed Swin-UNet cGAN framework, a comprehensive ablation study was designed focusing on 3 critical components. First, model sensitivity to the magnitude of the adversarial loss was evaluated by testing varying adversarial weights ranging from 0.0001 to 0.1 to determine the optimal balance between pixel-wise segmentation accuracy and adversarial shape refinement. Second, to assess the impact of training stability, a constant weight schedule was compared against a linear warm-up strategy, in which adversarial weights increased linearly from zero to the target value over the training epochs. Third, to identify the most effective objective function for segmenting fine cartilage structures within the adversarial framework, standard BCE loss alone was compared with a hybrid objective combining BCE and Dice loss.

### Statistical Analysis

Statistical analyses were performed using R (version 4.3.1; R Foundation for Statistical Computing). For multiple comparisons of the results, the normality of the data distribution was first assessed. Data following a normal distribution were analyzed using repeated measures ANOVA, whereas nonnormally distributed data were analyzed using the Friedman test. Post hoc comparisons were conducted using the Bonferroni correction. Inter- and intrarater reliability were evaluated using the DSC. A *P* value of less than .05 was considered statistically significant.

## Results

### Cartilage Segmentation Results

DSC, mean IoU, ASSD, and HD95 results for femoral and tibial cartilage segmentation are summarized in Table 1.

**Table 1:** Knee cartilage segmentation results.

Structure and metric		Model, mean (SD)					*P* value	
		UNet	UNet cGAN^a^	Swin-UNet	Proposed (Swin-UNet cGAN)			
**Femur**								<.001
	DSC^b^ (%)	78.6 (3.1)^c^	80.2 (1.2)^d^	76.5 (4.5)^e^	82 (1.5)^f^			
	IoU^g^ (%)	69.4 (3.9)^c^	69.9 (1.8)^c^	65.3 (5.2)^e^	71.9 (2.1)^d^			
	ASSD^h^ (mm)	1.34 (0.32)^e^	0.24 (0.05)^d^	0.58 (0.17)^c^	0.22 (0.08)^d^			
	HD95^i^ (mm)	5.14 (1.64)^e^	1.25 (0.21)^d^	2.94 (0.96)^c^	1.31 (0.27)^d^			
**Tibia**								<.001
	DSC (%)	79.2 (6.3)^cd^	80 (1.8)^c^	70.5 (8.6)^e^	81 (1.7)^d^			
	IoU (%)	69.7 (7.3)^cd^	70.1 (2.5)^c^	59.5 (8.5)^e^	71.1 (2.1)^d^			
	ASSD (mm)	1.05 (1.46)^e^	0.32 (0.27)^d^	0.83 (0.83)^c^	0.23 (0.15)^f^			
	HD95 (mm)	3.62 (5.62)^e^	1.46 (1.13)^d^	3.33 (2.89)^c^	1.25 (0.53)^f^			

^a^cGAN: conditional generative adversarial network.

^b^DSC: Dice similarity coefficient.

^c-f^Footnotes c, d, e, and f indicate significant differences between models based on post hoc pairwise comparisons (*P*<.05). Superscript letters follow the journal style (alphabetical order by first appearance) and do not represent a rank order of performance. Means sharing the same footnote are not significantly different.

^g^IoU: intersection over union.

^h^ASSD: average symmetric surface distance.

^i^HD95: 95th percentile Hausdorff distance.

For femoral cartilage, the baseline UNet achieved a mean DSC of 78.6% (SD 3.1%) and an IoU of 69.4% (SD 3.9%), with an ASSD of 1.34 (SD 0.32) mm and an HD95 of 5.14 (SD 1.64) mm. Adding adversarial training in UNet cGAN slightly improved overlap (DSC 80.2%, SD 1.2%; IoU 69.9%, SD 1.8%; *P*<.001 vs UNet) and markedly reduced surface errors (ASSD 0.24, SD 0.05 mm; HD95 1.25, SD 0.21 mm; *P*<.001 vs UNet). Swin-UNet without adversarial training underperformed both UNet and UNet cGAN in overlap metrics (DSC 76.5%, SD 4.5%; IoU 65.3%, SD 5.2%) and showed only intermediate surface accuracy (ASSD 0.58, SD 0.17 mm; HD95 2.94, SD 0.96 mm). The proposed Swin-UNet cGAN achieved the best femoral cartilage segmentation, with a DSC of 82% (1.5%) and an IoU of 71.9% (SD 2.1%; *P*<.001 vs all other models), and significantly lower ASSD and HD95 than UNet and Swin-UNet (*P*<.001), while maintaining surface accuracy comparable to UNet cGAN.

For tibial cartilage, the baseline UNet yielded a DSC of 79.2% (SD 6.3%) and an IoU of 69.7% (SD 7.3%), with an ASSD of 1.05 (SD 1.46) mm and an HD95 of 3.62 (SD 5.62) mm. UNet cGAN preserved similar overlap (DSC 80%, SD 1.8%; IoU 70.1%, SD 2.5%) but substantially reduced surface errors (ASSD 0.32, SD 0.27 mm; HD95 1.46, SD 1.13 mm). Swin-UNet again showed the weakest performance (DSC 70.5%, SD 8.6%; IoU 59.5%, SD 8.5%; ASSD 0.83, SD 0.83 mm; HD95 3.33, SD 2.89 mm). The proposed Swin-UNet cGAN yielded the highest overall tibial performance, achieving a DSC of 81% (SD 1.7%) and an IoU of 71.1% (SD 2.1%). While it showed significantly higher overlap than both Swin-UNet and UNet cGAN (*P<.*001), the difference compared with the standard UNet was not statistically significant. Importantly, it yielded the lowest ASSD (0.23, SD 0.15 mm) and HD95 (1.25, SD 0.53 mm) among all models (*P*<.001 vs all baselines). Taken together, the progression from UNet to UNet cGAN, Swin-UNet, and Swin-UNet cGAN effectively serves as an ablation series that disentangles the contributions of adversarial training and the Swin-Transformer backbone.

The proposed Swin-UNet cGAN achieved highly efficient inference performance. On an NVIDIA GeForce RTX 4090, the average inference time was 16.9 ms per 2D slice, culminating in a total processing time of 1.68 seconds per patient volume. This corresponds to a throughput of approximately 59 slices per second, demonstrating the model’s suitability for near-real-time clinical workflows.

### Qualitative Comparison of Segmentation Outputs

**Figure 2:**
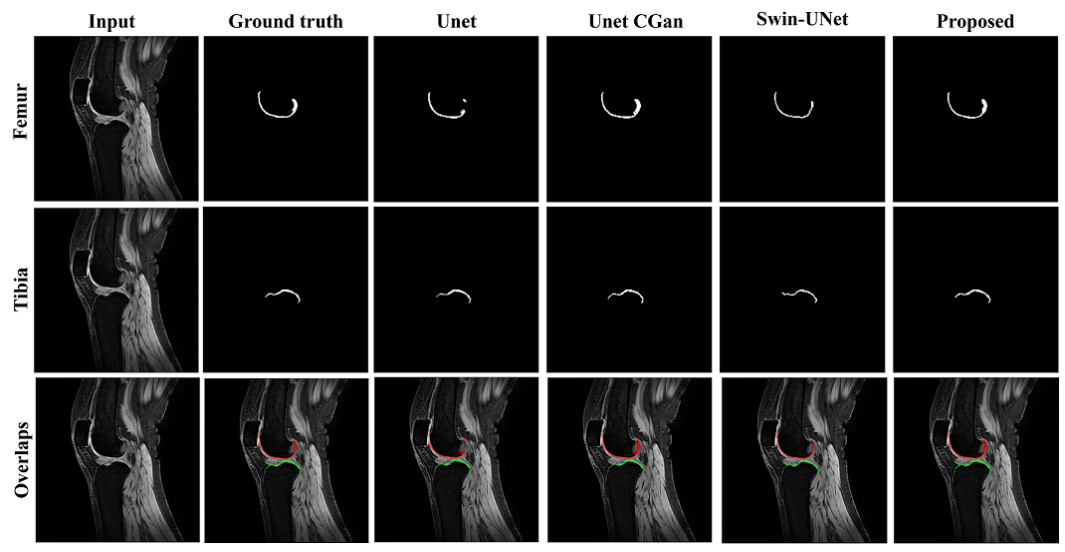
Qualitative comparison results of our proposed model and baseline models on the knee cartilage segmentation dataset.

Figure 2 illustrates qualitative segmentation outputs of the models on unseen test data. The rows correspond to the femur, tibia, and overlaps (segmentation masks overlaid on the input MRI), respectively. The columns display the Input image, ground truth, and predictions from UNet, UNet cGAN, Swin-UNet, and our proposed Swin-UNet cGAN (Ours). As shown in the “Overlaps” row, the proposed model produces the most visually accurate segmentation for both bone and cartilage compared with the baseline models.

### Reliability of Manual Segmentation

Inter- and intrarater agreement calculations for DSC and IoU scores comparing overlap between labeled masks showed high reliability on the original dataset. For intrarater agreement, DSCs for rater 1 and rater 2 averaged 98.9% and 99%, respectively, across femur and tibia. The IoU scores also were high at 98% and 98.3%, demonstrating excellent internal consistency for labeling MRI data. For interrater agreement, the DSC was 95.5% and the mean IoU was 91.9%, indicating greater than 90% agreement between the 2 raters.

### External Validation

Quantitative evaluation results on the external validation dataset are summarized. The proposed model demonstrated consistent segmentation performance across both femoral and tibial structures. For the femur, the model achieved a DSC of 73.1% (SD 6.9%) and an IoU of 65% (SD 6.9%). In terms of boundary delineation accuracy, ASSD was 1.30 (SD 0.70) mm and the HD95 was 5.91 (SD 2.93) mm. For the tibia, the model yielded a DSC of 69.7% (SD 7.4%) and an IoU of 62.6% (SD 7.2%). The distance-based metrics showed similar precision to the femur, with an ASSD of 2.16 (SD 0.94) mm and an HD95 of 7.18 (SD 2.64) mm.

### Ablation Study on Model Component

A comprehensive ablation study was conducted to optimize the Swin-UNet cGAN configuration, as summarized in Table 2. First, regarding the adversarial weight, a value of 0.0001 yielded the best performance, whereas increasing the weight led to instability and performance degradation. Second, the linear warm-up strategy outperformed the constant schedule, improving tibial metrics and training stability. Although femoral surface errors were marginally higher with warm-up, this trade-off was negligible given the overall gains. Finally, BCE loss alone outperformed the combined BCE + Dice loss, likely because the discriminator sufficiently enforces global shape constraints, making the additional geometric guidance from Dice loss redundant.

**Table 2:** Ablation study results of the proposed Swin-UNet conditional generative adversarial network framework analyzing the impact of adversarial weight, scheduling strategies, and segmentation loss functions on segmentation performance.

Component and configuration		Femoral cartilage, mean (SD)					Tibial cartilage, mean (SD)			
		DSC^a^ (%)	IoU^b^ (%)	ASSD^c^ (mm)	HD95^d^ (mm)	DSC (%)		IoU (%)	ASSD (mm)	HD95 (mm)
**Adversarial weight**										
	0.0001	81.67 (1.3)	71.52 (1.9)	0.21 (0.05)	1.25 (0.2)	80.55 (1.9)		70.62 (2.3)	0.27 (0.2)	1.35 (0.75)
	0.001	80.01 (3.06)	69.58 (3.4)	0.24 (0.05)	1.30 (0.22)	78.63 (2.05)		68.47 (2.5)	0.38 (0.26)	1.64 (0.99)
	0.01	76.75 (2.5)	65.53 (3.03)	0.3 (0.05)	1.41 (0.2)	69.61 (3.3)		58.25 (3.8)	0.83 (0.32)	2.9 (1.01)
	0.1	55.48 (2.6)	43.02 (2.3)	2.21 (0.53)	3.25 (1.22)	42.92 (3.14)		30.6 (2.8)	3.51 (1.03)	9.79 (3.43)
**Scheduling**										
	Constant	81.67 (1.3)	71.52 (1.9)	0.21 (0.05)	1.25 (0.2)	80.55 (1.9)		70.62 (2.3)	0.27 (0.2)	1.35 (0.75)
	Warm-up	82 (1.5)	71.9 (2.1)	0.22 (0.08)	1.31 (0.27)	81 (1.7)		71.1 (2.1)	0.23 (0.15)	1.25 (0.53)
**Segmentation loss**										
	BCE^e^	82 (1.5)	71.9 (2.1)	0.22 (0.08)	1.31 (0.27)	81 (1.7)		71.1 (2.1)	0.23 (0.15)	1.25 (0.53)
	BCE + Dice	81.28 (1.5	70.88 (2.2)	0.27 (0.04)	1.43 (0.2)	79.17 (2.28)		68.84 (2.8)	0.39 (0.32)	1.73 (1.3)

^a^DSC: Dice similarity coefficient.

^b^IoU: intersection over union.

^c^ASSD: average symmetric surface distance.

^d^HD95: 95th percentile Hausdorff distance.

^e^BCE: binary cross-entropy.

## Discussion

The most important finding of this study is that the proposed Swin-UNet cGAN model provided overall strong knee cartilage segmentation compared with the baseline UNet, UNet cGAN, and Swin-UNet models. Specifically, our model achieved higher DSC and IoU while maintaining tibial mask overlap comparable to UNet, and demonstrated markedly lower ASSD and HD95 while maintaining femoral surface accuracy comparable to UNet cGAN. These findings confirm our hypothesis that the Swin-UNet cGAN model achieves the highest performance in segmenting femoral and tibial cartilage.

Our results demonstrate that combining hierarchical Swin self-attention with adversarial refinement effectively captures the fine-grained pixel information needed for segmentation of knee cartilage. By embedding the Swin-UNet generator into a cGAN framework, we achieve superior cartilage delineation: the hierarchical self-attention mechanism—leveraging windowed self-attention rather than relying solely on skip connections—accurately traces cartilage boundaries and anatomically curved regions. Moreover, the Swin transformer backbone constructs a multiscale feature hierarchy through patch merging and expansion, enabling the network to integrate both detailed structural cues and broader contextual information more effectively than CNN-based encoders such as UNet.

For femoral cartilage, the proposed Swin-UNet cGAN model showed a statistically significant improvement over all baseline models, including UNet, UNet cGAN, and Swin-UNet, with higher DSC and IoU and markedly lower ASSD and HD95 (*P*<.05), while maintaining comparable surface accuracy to UNet-cGAN. Notably, for tibial cartilage, UNet-cGAN showed a statistically significant improvement over plain UNet, whereas the difference between the proposed Swin-UNet cGAN and UNet, although numerically larger, did not reach statistical significance. This finding is likely related to the relatively large intercase variability of the UNet baseline and the limited sample size, which reduce statistical power to detect small additional gains. Nevertheless, the proposed model consistently achieved the highest mean performance and the lowest ASSD and HD95, indicating the best overall balance between volumetric overlap and surface accuracy. From a clinical perspective, MRI-based PSI relies on accurate 3D surface reconstruction rather than volumetric overlap alone. Therefore, the substantial reductions in ASSD and HD95 achieved by the proposed Swin-UNet cGAN are particularly meaningful, as they indicate more precise cartilage boundaries that are directly relevant for the design and fit of PSI cutting guides.

An interesting finding of this study is that the plain Swin-UNet achieved lower DSC and IoU than UNet for both femoral and tibial cartilage. This may be explained by the inherent structural advantages of convolutional architectures such as UNet, which are highly effective at extracting local image features and can be trained stably even with relatively small datasets. In contrast, transformer-based architectures such as Swin-UNet are designed to capture long-range global context but typically require large-scale training data to fully learn these relationships. Under the limited knee MRI dataset used in this study, plain Swin-UNet appears to have suffered from overfitting and unstable convergence, resulting in lower performance (76.5%) than UNet (79.2%).

In this context, the effectiveness of the proposed Swin-UNet cGAN becomes more evident. As shown in Table 1, although the standalone Swin-UNet performed poorly, its performance markedly improved to 82% when used as the generator within a cGAN framework, surpassing that of UNet (79.2%). This suggests that adversarial learning, through the discriminator, imposes strong anatomical shape constraints on the generator, thereby compensating for the data dependency of the transformer architecture and enabling more accurate cartilage segmentation.

The disproportionate improvement in surface metrics (ASSD and HD95) compared with overlap metrics (DSC and IoU) can be attributed to the fundamental differences in their sensitivity. While DSC and IoU measure volumetric overlap and are relatively insensitive to minor boundary fluctuations, distance-based metrics such as ASSD and HD95 directly quantify contour delineation precision. Adversarial training in the proposed framework explicitly optimizes boundary realism through the discriminator, thereby yielding substantial gains in boundary precision even when volumetric improvements appear modest.

To benchmark our method against the current state of the art, we evaluated nnUNet on the same dataset (Table S1 in Multimedia Appendix 1). While nnUNet provided competitive volumetric overlap, the Swin-UNet cGAN achieved statistically significant improvements in both DSC (*P*=.02) and IoU (*P*=.04). Crucially, nnUNet struggled with boundary precision in severe osteoarthritis cases, yielding high HD95 values (2.55 mm and 2.75 mm for femur and tibia, respectively). In contrast, the proposed model markedly reduced surface errors, achieving HD95 values of 1.31 mm and 1.25 mm (*P*<.001) and significantly lower ASSD (*P*<.001). This demonstrates that the adversarial framework offers superior robustness in capturing the fine, irregular boundaries of degenerated cartilage compared with the self-configuring UNet approach.

Previous research by Chen et al [20] demonstrated that using Segmentation of Knee Images 2010 (SKI10) data, their model achieved a 1% to 2% DSC improvement over nnUNet, with results of 89% for femur cartilage and 88% for tibia cartilage. Guo et al [21] created a dataset of 700 MRI scans by pooling data from Osteoarthritis Initiative (OAI; n=200), fastMRI (n=175), SKI10 (n=100), and an internal hospital database (n=225). They trained 4 distinct DL models—UNet, UNet++, residual UNet (ResUNet), and transformer UNet (TransUNet)—on this dataset with corresponding labeled cartilage. Among these, TransUNet showed superior performance, achieving DSCs of 82.3% for femur cartilage and 80.3% for tibia cartilage. Further innovation came from Gaj et al [11], who developed a DL model for cartilage and meniscus segmentation by integrating a conditional GAN with UNet. This approach led to notable performance improvements, with femur cartilage segmentation increasing from 84.2% to 89.7% (a greater than 5% gain) compared with traditional UNet. For tibia cartilage, the method increased medial cartilage segmentation from 77% to 86.1% and lateral cartilage from 86.2% to 91.8%, using OAI data. Gaj et al’s [11] work clearly illustrates that cGANs can significantly enhance the capabilities of established models like UNet. A quantitative benchmarking table summarizing the datasets, methods, and metrics of these existing studies is presented in Table 3.

**Table 3:** Comparison of segmentation performance with previous methods.

Study (year) and method		Dataset	Performance
**Liu et al [22] (2019)**			
	SUSAN	SKI10^a^ (n=50) and internal (n=120)	Femur (DSC^b^=81%; ASSD^c^=0.65 mm) Tibia (DSC=75%; ASSD=0.73 mm)
**Gaj et al [11] (2020)**			
	UNet cGAN^d^	OAI^e^ (n=176)	Femur (DSC=89.7%) Tibia (DSC=91.8%)
**Chen et al [20] (2022)**			
	3D DNN^f^ with adversarial loss	SKI10 (n=100)	Femur (DSC=89%) Tibia (DSC=88%)
**Guo et al [21] (2024)**			
	TransUNet^g^	OAI (n=200), fastMRI (n=175), SKI10 (n=100), and internal (n=225)	Femur (DSC=82.3%) Tibia (DSC=80.3%)
**Wang et al [10] (2025)**			
	PA-UNet^h^ (2D)	OAI-ZIB^i^ (n=507)	Femur (DSC=88.8%; HD95^j^=9.1 mm) Tibia (DSC=82.7%; HD95=11.7 mm)
	PA-UNet^h^ (2D)	SKI10 (n=100)	Femur (DSC=78.2%; HD95=16.4 mm) Tibia (DSC=71.6%; HD95=18.7 mm)
**Proposed framework**			
	Swin-UNet cGAN	Internal (n=232)	Femur (DSC=82%; ASSD=0.22 mm; HD95=1.31 mm) Tibia (DSC=81%; ASSD=0.23 mm; HD95=1.25 mm)

^a^SKI10: Segmentation of Knee Images 2010.

^b^DSC: Dice similarity coefficient.

^c^ASSD: average symmetric surface distance.

^d^cGAN: conditional generative adversarial network.

^e^OAI: Osteoarthritis Initiative.

^f^DNN: deep neural network.

^g^TransUNet: transformer UNet.

^h^PA-UNet: patch attention UNet.

^i^OAI-ZIB: Osteoarthritis Initiative–Zuse Institute Berlin.

^j^HD95: 95th percentile Hausdorff distance.

Beyond spatial overlap assessed by DSC, evaluating boundary precision is crucial for clinical applications such as PSI. Liu et al [22] reported ASSD values of 0.65 mm and 0.73 mm for femoral and tibial cartilage, respectively, using their SUSAN framework. Most recently, Wang et al [10] applied a patch attention UNet (PA-UNet) to the Osteoarthritis Initiative–Zuse Institute Berlin (OAI-ZIB) and SKI10 datasets. Although they achieved competitive DSCs, they reported HD values ranging from 9.1 mm to 18.7 mm, indicating potential challenges in handling outlier boundary pixels.

In this study, the proposed Swin-UNet cGAN demonstrated high surface accuracy, achieving ASSD values of 0.22 mm and 0.23 mm and HD95 values of 1.31 mm and 1.25 mm for femoral and tibial cartilage, respectively. Although direct quantitative comparison is challenging due to differences in datasets, these low error rates suggest that integrating the Swin Transformer with adversarial learning effectively refines segmentation boundaries and may offer superior precision for clinical workflows.

A common limitation observed in prior studies is the use of mixed datasets that combine MRI scans from both healthy individuals and patients without explicitly accounting for osteoarthritis progression. Because knee MRI segmentation is most frequently applied in the context of arthroplasty, we trained our DL model using MRI data from patients diagnosed with Kellgren-Lawrence Grades 3-4 who were already scheduled for TKA. This targeted methodology ensures that our model’s findings are highly relevant and directly applicable to real-world clinical scenarios, particularly for patients with advanced osteoarthritis, for whom accurate segmentation is critical for surgical planning and improved patient outcomes.

In addition, a recently published study demonstrated that, compared with training on mixed datasets containing both healthy participants and patients, training primarily on patient data reduced femoral cartilage DSC from 88.8% to 78.2% and tibial cartilage DSC from 82.7% to 71.6% [10].

In terms of clinical relevance, our results are expected to bring significant advancements not only in quantitative diagnostics during knee arthroscopy but also in computer-assisted surgery, including robotic-assisted TKA. Most contemporary robotic‐assisted procedures are predicated almost entirely on image‐based workflows using CT [23]. However, the patient-specific model is generated exclusively from CT data and includes only the bony anatomy; thus, all registration landmarks must be acquired directly on bone during robot-assisted TKA. Intraoperatively, this requirement necessitates a sharp probe to penetrate the overlying cartilage until the underlying bone surface is reached, ensuring accurate alignment. However, an MRI-derived model inherently preserves the cartilage in its segmentation, facilitating a more straightforward and intuitive registration process.

The clinical impact of incorporating cartilage information has been quantified in several comparative studies [24,25]. Additionally, recent meta-analyses have confirmed that MRI-based PSI demonstrates fewer alignment outliers and superior accuracy compared with CT-based techniques, especially in knees with irregular or worn cartilage [26,27]. The practical implications of the inability of CT to visualize cartilage have led to compensatory measures, such as assuming uniform cartilage thickness or manually removing residual cartilage intraoperatively, potentially compromising surgical precision. In addition, recent studies have shown that CT-based PSI can result in inaccurate alignment in varus deformity knees [28]. According to their study, the authors recommended adding approximately 2 mm of cartilage thickness to the planned lateral tibial resection value in CT-based PSI workflows for patients with varus deformity [28]. However, because cartilage thickness can vary substantially between individuals and across different regions of the joint, artificially increasing the resection thickness by a fixed 2 mm only on the lateral side may introduce design challenges and potential inaccuracies in PSI planning. In contrast, MRI-based PSI directly incorporates patient-specific cartilage thickness, thereby eliminating the need for such heuristic adjustments and potentially overcoming these limitations. Our Swin-UNet cGAN model directly addresses these well-documented limitations by providing accurate, automated segmentation of both femoral and tibial cartilage. This advancement has the potential to eliminate the alignment errors associated with CT-based PSI, representing a significant step toward more precise, reproducible surgical outcomes in TKA.

This study has several limitations. First, we performed segmentation of only the knee joint (distal femur and proximal tibia) cartilage. Future research should expand to include the ligaments and menisci. However, the MRI protocol optimized for PSI fabrication provides a suboptimal evaluation of ligamentous and meniscal structures. Second, the primary training and internal test datasets were acquired on a single 3.0 T MRI system (uMR 770; United Imaging Healthcare) using 1 PSI-optimized 3D T2*-weighted gradient-echo sequence. Although this relatively homogeneous acquisition protocol reduces within-study variability, it may limit the generalizability of our model to other scanner vendors, field strengths, coil configurations, and cartilage-focused imaging protocols beyond those used in this study. External validation was performed on a separate 3.0 T MRI system (SIGNA Premier; GE Healthcare), where segmentation performance was slightly lower than on the internal test set, likely reflecting a degree of domain shift related to differences in slice thickness (2 mm for the external dataset vs 1 mm for the internal dataset), with reduced through-plane resolution and increased partial-volume effects. Nevertheless, the model maintained reasonably high performance on this external cohort, indicating partial robustness to protocol variation. Larger multicenter studies with more heterogeneous MRI protocols and scanner types, together with additional training on datasets acquired with thicker slices and/or explicit domain-adaptation strategies, are needed to further narrow this performance gap and fully establish robustness across diverse clinical environments. Third, the current model uses a downsampled input resolution of 224 × 224 and performs slice-wise 2D segmentation, without explicitly leveraging full 3D spatial context, which is important for volumetric MRI analysis. This resolution choice was primarily driven by the need to manage the high memory complexity of the Swin Transformer backbone within a dual-network GAN framework during training, rather than solely by inference speed constraints. We acknowledge that this downsampling results in an approximate pixel spacing of 0.89 mm, which is relatively coarse compared with thin cartilage structures and may limit the capture of extremely fine details. However, contemporary PSI design workflows for TKA, including most commercial systems, are typically based on sagittal 2D cartilage contours that are subsequently reconstructed into 3D surfaces, making a 2D architecture practically compatible with current clinical and industrial pipelines. While our use of surface-based metrics (ASSD and HD95) provides an indirect assessment of 3D geometric accuracy, future work should extend the framework to 2.5D or 3D architectures with high resolution and directly evaluate interslice consistency and PSI template fit. Fourth, although our proposed model is more advanced than those in previous studies, it exhibited a slightly lower DSC. However, as noted above, this limitation can be attributed to our dataset, which consists of patients (Kellgren-Lawrence grades 3-4) awaiting TKA, where extensive cartilage damage hinders segmentation compared with that in a previous study [11]. Nonetheless, we demonstrated that our Swin-UNet cGAN outperforms the UNet cGAN used in previous studies, even when evaluated on this highly challenging dataset. Future work will explore self-supervised pretraining or advanced augmentation to further improve data efficiency and push performance on small‐sample domains.

The Swin-UNet cGAN integrates hierarchical shifted-window attention into its UNet-style transformer generator and uses a pixel-level CNN discriminator to refine outputs, enabling overall more effective segmentation of fine cartilage structures than the standard UNet, the UNet cGAN, and the Swin-UNet, and this advantage was consistently preserved on an external validation dataset. Clinically, this MRI-based segmentation approach has the potential to address key limitations of current CT-based PSI and robotic-assisted TKA workflows by providing accurate visualization of femoral and tibial cartilage, enabling more anatomically faithful preoperative models, reducing alignment errors related to unmodeled cartilage thickness, and supporting more intuitive intraoperative registration on the articular surface.

## Appendix

Multimedia Appendix 1 Quantitative comparison of segmentation performance between nnUNet and the proposed Swin-UNet cGAN.

## Abbreviations

ASSD: average symmetric surface distance
BCE: binary cross-entropy
cGAN: conditional generative adversarial network
CNN: convolutional neural network
CT: computed tomography
DL: deep learning
DSC: Dice similarity coefficient
GAN: generative adversarial network
HD: Hausdorff distance
HD95: 95th percentile Hausdorff distance
IoU: intersection over union
IRB: Institutional Review Board
MRI: magnetic resonance imaging
OAI: Osteoarthritis Initiative
PSI: patient-specific instrument
ResUNet: residual UNet
SKI10: Segmentation of Knee Images 2010
TE: echo time
TKA: total knee arthroplasty
TR: repetition time
TransUNet: transformer UNet

## Data Availability

The datasets generated or analyzed during this study are not publicly available due to institutional review board restrictions but are available from the corresponding author on reasonable request.
