# Supplementary material for: Enhanced Magnetic Resonance Imaging–Based Knee Cartilage Segmentation Using a Swin-UNet Conditional Generative Adversarial Network: Development and Validation Study
Source: JMIR Med Inform. 2026 Mar 2;14:e86155. doi: 10.2196/86155 (PMC12993272; doi:10.2196/86155)
Supplement: Multimedia Appendix 1 [file medinform_v14i1e86155_app1.docx]

**Table S1.** Quantitative comparison of segmentation performance between nnUNet and the proposed Swin-UNet cGAN

|  | | Model | | P-value |
| --- | --- | --- | --- | --- |
|  |  | nnUNet | Proposed  (Swin-UNet CGAN) |  |
| Femur | DSC (%) | 80.3 ± 3.4 | 82.0 ± 1.5 | .02 |
|  | IoU (%) | 70.2 ± 4.3 | 71.9 ± 2.1 | .04 |
|  | ASSD (mm) | 0.61 ± 0.18 | 0.22 ± 0.08 | *<*.001 |
|  | HD95 (mm) | 2.55 ± 1.05 | 1.31 ± 0.27 | *<*.001 |
| Tibia | DSC (%) | 77.4 ± 9.1 | 81.0 ± 1.7 | .007 |
|  | IoU (%) | 67.1 ± 9.6 | 71.1 ± 2.1 | .004 |
|  | ASSD (mm) | 0.73 ± 0.90 | 0.23 ± 0.15 | *<*.001 |
|  | HD95 (mm) | 2.75 ± 3.38 | 1.25 ± 0.53 | *<*.001 |
